# *HNF1A* gene *p.I27L* is associated with early-onset, maturity-onset diabetes of the young-like diabetes in Turkey

**DOI:** 10.1186/s12902-019-0375-2

**Published:** 2019-05-20

**Authors:** Selvihan Beysel, Nilnur Eyerci, Ferda Alparslan Pinarli, Muhammed Kizilgul, Ozgur Ozcelik, Mustafa Caliskan, Erman Cakal

**Affiliations:** 1 Department of Endocrinology and Metabolism, Diskapi Yildirim Beyazit Teaching and Training Research Hospital, Ankara, Turkey; 2 Department of Medical Biology, Baskent University, Ankara, Turkey; 3 Department of Genetic Research, Diskapi Yildirim Beyazit Teaching and Research Hospital, Ankara, Turkey; 4 Department of Endocrinology and Metabolism, Afyonkarahisar Saglik Bilimleri University, Afyonkarahisar, Turkey

**Keywords:** *P.I27L*, *P.A98V*, *HNF1A* gene, Diabetes

## Abstract

**Background:**

The molecular basis of the Turkish population with suspected maturity-onset diabetes of the young (MODY) has not been identified. This is the first study to investigate the association between *HNF1A*-gene single-nucleotide polymorphisms (SNPs) and having early-onset, MODY-like diabetes mellitus in the Turkish population.

**Methods:**

All diabetic patients (*N* = 565) who presented to our clinic between 2012 and 2015 with a clinical suspicion of MODY were included in the study. Analysis of *HNF1A, HNFB, HNF4A, GCK* gene mutations was performed using real-time polymerase chain reaction sequencing. After genetic analysis, diabetics (*n* = 46) with *HNF1A, HNF1B, HNF4A, GCK* gene mutations (diagnosed as MODY) and diabetics (*n* = 30) with *HNF1B, HNF4A, GCK* gene SNPs were excluded. Patients with early-onset, MODY-like diabetes (*n* = 486) and non-diabetic controls (*n* = 263) were included. Genetic analyses for the *HNF1A* gene *p.S487 N* (rs2464196), *p.A98V* (rs1800574) and *p.I27L* (rs1169288) SNPs were performed using Sanger-based DNA sequencing among the control group.

**Results:**

*p.S487 N* and *p.A98V* was similar between the diabetics and controls in dominant and recessive models with no association (each, *p* > 0.05). *p.I27L* GT/TT carriers (GT/TT vs. GG, OR = 1.68, 95% CI: [1. 21-2.13]; *p* = 0.035) and *p.I27L* TT carriers had increased risk of having MODY-like diabetes (GT/GG vs. TT, OR = 1.56, 95% CI: [1. 14-2.57]; *p* = 0.048). Family inheritance of diabetes was significantly more common in patients with the p.I27L TT genotype. The *p.I27L* SNP was modestly associated with having diabetes after adjusting for body mass index and age (*β* = 1.45, 95% CI: [1. 2-4.2]; *p* = 0.036).

**Conclusions:**

The *HNF1A* gene *p.I27L* SNP was modestly associated with having early-onset, MODY-like diabetes in the Turkish population. *HNF1A* gene p.I27L SNP might contribute to age at diabetes diagnosis and family inheritance.

**Electronic supplementary material:**

The online version of this article (10.1186/s12902-019-0375-2) contains supplementary material, which is available to authorized users.

## Background

Hepatocyte nuclear factor 1A (HNF1A) is a transcription factor that has a role in the development and function of pancreas ß-islet cells. In the developmental stage, both endocrine and exocrine cells of the pancreas have HNF1A expression [1]. HNF1A is necessary for insulin secretion in response to glucose [2–4]. The *HNF1A* gene has been identified in both monogenic and polygenetic diabetes. Rare mutations of the *HNF1A* gene cause a monogenic form of diabetes as Type 3 maturity-onset-diabetes of the young (MODY3) [1]. The *HNF1A* gene contributes to the pathogenesis of Type 2 diabetes mellitus (T2DM). *HNF1A* gene single nucleotide polymorphisms (SNPs) were modestly associated with Type 2 diabetes mellitus (T2DM) and glycemic features in different populations [5–7]. *HNF1A* SNPs were associated with impaired insulin secretion [8, 9]. *HNF1A* gene SNPs (*p.I27L, p.A98V* and *p.S487 N*) were inconsistently associated with impaired glucose tolerance and having diabetes [8–14]. Some young people with diabetes have atypical features such as insulin resistance or a need for insulin treatment. However, these features are not similar to T2DM. These non-obese adults have early-onset, MODY-like diabetes. Monogenic MODY has not been confirmed in patients with early-onset, MODY-like diabetes through genetic analysis [15]. The genetic basis of early-onset, non-monogenic diabetes is not yet known. The aim of this study was to obtain the effects of *HNF1A* gene SNPs on developing MODY-like diabetes. This is the first study to investigate the association between *HNF1A* gene SNPs rs1169288 (encoding *HNF1A p.Ile27Leu*), rs1800574 (encoding *HNF1A p.Ala98Val*) and rs2464196 (encoding *HNF1A p.Ser486Asn*), and having early-onset, MODY-like diabetes in the Turkish population.

## Methods

### Patients

In our study, none of the control subjects (*n* = 263) had diabetes. All patients with diabetes (*n* = 486) met the criteria for the diagnosis of MODY. Subjects with a clinical suspicion of MODY [16] (diagnosis of diabetes age below 25 years, positive family history including autosomal dominant inheritance in at least 2-3 generations, residual insulin secretion with normal C-peptide concentration and absence of B-cell autoimmunity) who presented to our hospital between 2012 and 2015 were included in the study. The inclusion criteria were as follows; patients with T2DM with C-peptide concentrations ≥0.3 nmol/L, negative anti-GAD antibodies, and age-at-onset below 25 years [2]. Patients with suspected MODY did not need insulin treatment for at least first 2 years after diagnosis and had no family history of T1DM [17]. Early or late-onset diabetes was identified by using age 45 years as a cut-off, as described in previous studies [2, 17]. If we selected control subjects from those whose mean age was below 28 years, some of these subjects would develop diabetes later in life. As a way of reducing the possibility of recruiting control subjects who might later develop T2DM, healthy-normoglycemic subjects with fasting glucose below 100 mg/dL and glycated hemoglobin (Hb1Ac) < 5.7%, who were aged ≥45 years and had no first-degree relatives or grandparents with T2DM were included in the control group [17]. Healthy controls without chronic disease such as diabetes, hypertension, renal and hepatic disease, were recruited from the outpatient clinic. Subjects with genetically confirmed MODY or T1DM were excluded [2]. Genetic analysis was performed for all patients (*n* = 565) in order to diagnose MODY. After genetic analysis, patients with diabetes (*n* = 46) who had *HNF1A, HNF1B, HNF4A, GCK* gene mutations were diagnosed as having MODY3, MODY5, MODY1, and MODY2 respectively. Thirty patients with diabetes had *HNF1B*, *HNF4A*, had *GCK* gene SNPs. Patients with diabetes with MODY and *HNF1B*, *HNF4A*, and *GCK* SNPs were excluded from the study. Finally, subjects without *HNF1A*, *HNF1B*, *HNF4A*, and *GCK* gene mutations and *HNF1B*, *HNF4A*, and *GCK* SNPs (*n* = 486) and non-diabetic healthy controls (*n* = 263) were included this study.

### Measurements

Fasting glucose, postprandial glucose, creatinine, HbA1c, triglycerides (TG), cholesterol, low-density lipoprotein cholesterol (LDL-C), high-density lipoprotein cholesterol (HDL-C), C-peptide, high-sensitivity (hs-CRP), anti-glutamic acid decarboxylase (GAD) antibody, anti-insulin antibody, anti-islet antibody, and urinary microalbuminuria concentrations were measured. Age and symptoms at onset of diabetes, diabetes treatment, and parental history of diabetes (first-degree relatives, mother or father) were recorded from all patients with diabetes. Body mass index (BMI) was calculated as weight (kg) / height (m^2^). BMI ≥ 30 kg/m^2^ was diagnosed as obesity. T2DM was diagnosed when plasma fasting glucose concentrations were > 125 mg/dL, casual or postprandial glucose levels were > 200 mg/dL or in the presence of current treatment with a hypoglycemic agent, according to the American Diabetes Association criteria [17]. Informed consent was obtained from all participants. This study was approved by Diskapi Yildirim Beyazit Training and Research Hospital Local Ethics Committee.

Genotyping and Statistical analysis is presented in Additional file 1.

## Results

The percentage of women (51.5% vs. 58.2%) and BMI value (27.88 ± 5.72 vs. 27.01 ± 3.29 kg/m^2^) was similar between the diabetics and controls (*p* > 0.05). The mean age of the controls was 49.18 ± 3.38 years. The mean age at onset of diabetes was 24.08 ± 4.82 years. The mean C-peptide concentration of the patients with diabetes was 2.47 ± 1.79 nmol/L. Fasting glucose, HbA1c, TG, cholesterol, LDL-C concentrations were higher among the diabetics compared with the controls (*p* < 0.05, Table 1). *HNF1A* gene *p.I27L* rs1169288 and *p.A98V* rs1800574 SNPs were consistent with the Hardy-Weinberg equilibrium (HWE), and *p.S487* rs2464196 were not consistent with the HWE (Table 2). *HNF1A* genotypes are shown in Table 3. Thefrequency of *p.S487 N* SNPs was similar between the diabetics and controls in the codominant model and dominant model and recessive model (*p* > 0.05, each)*. p.A98V* SNPs were similar between the diabetics and controls in the dominant model and recessive model (*p* > 0.05, each). The *p.A98V* TT genotype was higher in diabetics in the codominant model compared with the controls (TT vs. CC, OR = 1.35, 95% CI: [0.95–3.54]; *p* = 0.027). *HNF1A* gene *p.I27L* TT genotype was increased in diabetes (TT vs. GG, OR = 1.71, 95% CI: [1. 25-3.46]; *p* = 0.024) compared with the controls in the codominant model. *p.I27L* GT/TT carriers had increased 1.68 odds of having diabetes (GT/TT vs. GG, OR = 1.68, 95% CI: [1. 21-2.13]; *p* = 0.035) in the dominant model. *p.I27L* TT carriers had 1.56-fold increased odds of having diabetes (GT/GG vs. TT, OR = 1.56, 95% CI: [1. 14-2.57]; *p* = 0.048) in the recessive model. Clinical and biochemical characteristics did not differ between patients with diabetes with *p.I27L*, *p.S487 N*, and *p.A98V* SNPs and diabetics without the SNPs (*p* > 0.05). Onset of diabetes was 26.17 ± 7.4 years in *p.S487 N*, 25.58 ± 2.7 years in *p.A98V*, and 24.57 ± 5.2 years in *p.I27L* (*p* > 0.05). Parent diabetes (mother or father) was higher in the *p.I27L* TT genotype compared with the GG genotype (78.5 vs. 98.7%, *p* = 0.035). Diabetics with *p.I27L* TT genotype had higher triglyceride concentrations compared with diabetics with the GG genotype (*p* = 0.041) (Table 4). *HNF1A* gene *p.I27L* and *p.A98V* haplotypes were within Linkage Disequilibrium. *p.I27L* SNPs was modestly associated with having diabetes after adjusting for BMI and age (*β* = 1.45, 95% CI: [1. 2-4.2]; *p* = 0.036).

**Table 1:** Characteristics of subjects

	Controls (*n* = 263)	Diabetics (*n* = 486)	P
Women (%)	58.2	51.5	0.081
Parent diabetes (%)	30.4	96.1	**< 0.001**
Symptoms at the diagnosis (%)	–		–
Asymptomatic		58.1	
Diabetic symptom		32.5	
Gestational diabetes		8.1	
Diabetic complication		1.3	
Treatment (%)	–		–
Diet		20.1	
Oral antidiabetic		32.1	
İnsulin		47.8	
Age at diagnosis (year)^a^	–	24.08 ± 4.82	**–**
BMI (kg/m^2^)^a^	27.01 ± 3.29	27.88 ± 5.72	0.109
Systolic BP (mmHg)^a^	124.70 ± 11.15	125.31 ± 11.82	0.823
Diastolic BP (mmHg)^a^	76.64 ± 7.21	75.61 ± 7.86	0.374
Fasting glucose (mg/dl)^a^	80.68 ± 9.37	151.75 ± 74.12	**< 0.001**
Postprandial glucose (mg/dl)^a^	–	252.92 ± 110.97	**–**
LDL (mg/dl)^a^	96.90 ± 20.75	107.99 ± 36.98	**< 0.001**
TG (mg/dl)^a^	99.51 ± 51.64	204.46 ± 203.21	**< 0.001**
Cholesterol (mg/dl)^a^	159.18 ± 27.68	193.55 ± 87.46	**< 0.001**
HDL (mg/dl)^a^	51.33 ± 16.96	44.16 ± 14.07	**< 0.001**
Creatinine (mg/dl)^a^	0.88 ± 0.89	1.24 ± 8.91	0.018
HbA1c (%)^a^	5.31 ± 0.10	8.21 ± 2.41	**< 0.001**
TSH^a^	1.74 ± 1.01	2.78 ± 8.73	0.142
HsCRP^a^	3.30 ± 2.98	4.08 ± 3.96	0.101
C-peptide (nmol/L)^a^	–	2.47 ± 1.79	**–**
Microalbuminuria^a^	11.96 ± 13.65	94.86 ± 348.40	**< 0.001**

*BMI* body mass index, *HbA1c* hemoglobin A1c, *BP* blood pressure

^a^Student’s *t* test was used for normally distributed continuous variables or log-transformed variables between two groups

Data are shown as mean ± standard deviation (means ± SD) and percentage (%)

Bold represents the significant *p*-values

Categorical variables were analyzed with the Chi-square test or Fisher’s exact test, where appropriate

**Table 2:** Minor allele frequency of *HNF1A* gene SNPs

	Risk allele	MAF for study sample
I27L rs1169288	T	0.43
S487 N rs2464196	T	0.39
A98V rs1800574	T	0.09

*MAF* minor allele frequency

**Table 3:** Genotype analysis of HNF1A *gene* SNPs

	Controls, n	Diabetes, n	OR (95% CI)	P
***I27L*** rs1169288 (%)				
*Co-dominant Wild type GG	105	146		
Heterozygous GT	120	233	1.02 (0.57–1.78)	0.984
Homozygous TT	38	110	1.71 (1.25–3.46)	**0.024**
Dominant (GT + TT/GG)	158 vs 105	343 vs 146	1.68 (1. 21-2.13)	**0.035**
Recessive (TT/GT + GG)	38 vs 225	110 vs 379	1.56 (1. 14-2.57)	**0.048**
***S487 N*** rs2464196 (%)				
*Co-dominant Wild type CC	102	188		
Heterozygous CT	121	210	0.58 (0.35–1.39)	0.471
Homozygous TT	40	91	1.25 (0.57–2.75)	0.638
Dominant (CT + TT/CC)	161 vs 102	301 vs 188	1.01 (0.74–1.38)	0.938
Recessive (TT/CT + CC)	40 vs 223	91 vs 398	1.27 (0.84–1.91)	0.241
***A98V*** rs1800574 (%)				
*Co-dominant Wild type CC	208	411		
Heterozygous CT	52	64	1.26 (0.48–3.29)	0.676
Homozygous TT	3	14	1.35 (0.95–3.54)	**0.027**
Dominant model (CT + TT/CC)	55 vs 208	78 vs 411	0.71 (0.48–1.05)	0.089
Recessive model (TT/CT + CC)	3 vs 260	14 vs 475	2.55 (0.72–8.97)	0.130

*Co-dominat model was compared wild type, homozygous variant and heterozygous variant were compared

*DM* Diabetes mellitus, *OR* odds ratio, *CI* confidence interval

Data are shown as mean ± standard deviation (means ± SD) and percentage (%)

Bold represents the significant *p*-values

Categorical variables were analyzed with Chi-square test or Fisher’s exact test, where appropriate

Multiple logistic regression analysis and Fisher’s exact test were tested using models: dominant (major allele homozygotes vs heterozygotes + minor allele homozygotes), recessive (major allele homozygotes + heterozygotes vs minor allele homozygotes) and codominant (major allele homozygotes vs heterozygote and minor allele homozygotes vs major allele homozygotes)

**Table 4:** *HNF1A* gene *p.I27L* SNPs and clinical features in diabetics patients

	GG (wild) *n* = 146	GT *n* = 233	TT *n* = 110	P^a^ GG/GT	P^b^ GG/TT	P^c^ GT/TT
Age at the diagnosis (year)^a^	23.36 ± 9.51	20.89 ± 6.54	21.29 ± 9.78	0.845	0.653	0.958
Parent diabetes (%)	78.5	89.2	98.7	0.427	**0.035**	0.852
BMI (kg/m^2^)^a^	26.78 ± 6.02	27.81 ± 3.85	28.42 ± 4.70	0.871	0.852	0.990
Fasting glucose (mg/dl)^a^	151.12 ± 75.89	175.01 ± 98.35	154.01 ± 68.13	0.895	0.836	0.127
Postprandial glucose (mg/dl)^a^	239.43 ± 114.53	264.18 ± 100.37	276.18 ± 125.38	0.625	0.327	0.785
HbA1c (%)	7.85 ± 3.45	8.20 ± 6.75	8.47 ± 2.29	0.427	0.324	0.913
LDL (mg/dl)^a^	112.30 ± 37.61	106.09 ± 30.98	136.09 ± 45.65	0.358	0.339	0.249
TG (mg/dl)^a^	174.31 ± 175.80	197.91 ± 186.34	218.91 ± 276.52	0.258	**0.041**	0.377
Cholesterol (mg/dl)^a^	185.35 ± 96.84	189.27 ± 35.63	197.87 ± 44.90	0.957	0.924	0.847
HDL (mg/dl)^a^	46.22 ± 11.37	49.32 ± 17.93	43.28 ± 20.85	0.542	0.627	0.332
C-peptide (nmol/L)^a^	2.82 ± 2.28	2.15 ± 1.32	2.32 ± 1.51	0.246	0.351	0.513
Microalbuminuria	91.30 ± 357.93	96.42 ± 349.35	106.50 ± 320.85	0.792	0.650	0.838

^a^GG genotype vs GT genotype ^b^GG genotype vs TT genotype ^c^GT genotype vs TT genotype

Data are shown as mean ± standard deviation (means ± SD) and percentage (%)

Bold represents the significant *p*-values

*BMI* body mass index, *HbA1c* hemoglobin A1c

Student’s *t* test was used for normally distributed continuous variables or log-transformed variables between two groups

Categorical variables were analyzed with the Chi-square test or Fisher’s exact test, where appropriate

## Discussion

This case-control study showed that the *HNF1A* gene *p.I27L* SNP was modestly associated with having early-onset, MODY-like diabetes in the Turkish population. Family inheritance of diabetes was significantly more common in patients with the p.I27L TT genotype. The *HNF1A* gene p.I27L SNP might contribute to age at diabetes diagnosis and family inheritance.

In this study, we suggest that polygenic T2DM may show differences in age-related and family inheritance transmission for an associated monogenic form of diabetes. This is the first study to show the effect of the p.I27L genotype on modifying age at diagnosis in the Turkish population. We excluded monogenic diabetes modifier genes, which often include mutations, because we aimed to examine the influence of variations on polygenic diabetes. In our study, subjects with diabetes were non-obese and the onset of diabetes was early. A previous study showed that non-obese patients with early-onset diabetes were more susceptible to β-cell dysfunction as compared with old and obese individuals [18]. A modest association was found between *HNF1A* missense SNPs (*p.I27L*, *p.A98V*, and *p.S487 N*) and having late-onset T2DM in the European population [2–4]. In European ancestry, no association was shown between *HNF1A* gene SNPs and having late-onset T2DM [19], but a robust association was found when *p.A98V* SNPs were included [20]. Similar to our study, European ancestry reported that *p.I27L* and *p.A98V* SNPs were associated with having late-onset T2DM [12]. *p.I27L* GT/TT carriers had 1.68-fold increased odds of having diabetes, and *p.I27L* TT carriers had 1.5 6-fold increased odds of having diabetes in our study. Only the *p.I27L* variant was modestly associated with having diabetes and this relationship continued after adjusting BMI and age. There was no association between *p.S487 N* and *p.A98V* SNPs and early-onset T2DM. Similar to our report, a modest association was shown between *p.I27L*, *p.S487 N*, and *p.A98V* and having T2DM in the European population [19]. In agreement with our report, *p.I27L* was associated with having T2DM in non-obese French [21] and Finnish subjects [13]. A Chinese and Japanese meta-analysis reported that *p.I27L* was associated with having T2DM [18]. *HNF1A* gene *p.I27L* was associated with having late-onset T2DM in Brazilian [22] and Western Indian [23] overweight/obese subjects aged 51–60 years; however, this association was found in normal-weight Japanese subjects [11]. In line with our report, Holmkvist et al. determined that *p.I27L* was associated with having late-onset T2DM in overweight Scandinavian subjects aged over 60 years, and *p.A98V* was reported to decrease in vivo glucose-responsive to insulin secretion [2]. Chi et al. demonstrated that *p.I27L* has a modest role in β-cell dysfunction [10] and in insulin resistance [8, 24]. Consistent with our study, European population studies found a modest association between only *p.A98V* and having T2DM [3, 4]. A Danish study of Caucasians found *p.A98V* to be associated with decreased insulin secretion in healthy individuals [9], but this effect was balanced by increased insulin sensitivity [25]. *HNF1A* gene *p.A98V* was associated with having early-onset T2DM in Scandinavian [26] and Asian-Indian [27] individuals. *HNF1A p.A98V* was associated with having late-onset T2DM in Finnish but not in Chinese individuals [14]. Our study reported that the *p.A98V* TT genotype was higher compared with the GG genotype in diabetics, nevertheless, with no association.

Early-onset diabetes (19 years) was observed in Chinese p*.I27L + p.S487 N* carriers [28]. Yorifuji et al. reported that patients who were MODY-mutation–positive were younger and had a lower BMI percentile at diagnosis compared with mutation-negative patients in Japan [29]. A German-Austrian study reported that age at onset of diabetes (10.9 years) was found to be younger in *p.I27L + p.S487 N ± p.A98V* carriers, as compared with *HNF1A* mutation (14 years). Locke et al. reported that each p.I27L allele was associated with a 1.6-year decrease in age at diagnosis in patients with HNF1A-MODY [30]. Our study reported early onset of diabetes (24.08 ± 4.82 year) with no differences between *HNF1A* gene SNPs. Similar to our report, paternal diabetes was higher in *HNF1*A gene SNP carriers [31]. This study found that diabetes was higher in first-degree relatives (mother or father) of *p.I27L* homozygous TT carriers, suggesting a probability of significant familial transmission.

The *HNF1*A locus *p.I27L* is localized in the dimerization domain, *p.S487 N* is localized in the transactivation domain, and the *p.A98V* is localized in the DNA-binding domain [1, 22, 28]. *HNF1A* gene *p.I27L*, *p.A98V*, and *p.S487 N* variants reduce transcriptional activities of genes that have a role in glucose metabolism [2]. It was reported that *p.I27L + p.A98V* variations decreased transactivation activity on GLUT2 in HeLa cells more than *p.I27L* alone and *p.A98V* alone [2]. Decreased insulin secretion and ß-cell dysfunction was observed in p.I27L coexisting with p.487 N carrier (when p.A98V carrier included). This leads to developing diabetes [1, 2, 4, 24, 25, 31]. *HNF1A* controls ß-cell function by regulating target genes such as glucose transporter 2 (GLUT2), *HNF 4A*, collectrin, liver pyruvate kinase, and hepatocyte growth factor activator. *HNF1A* activity dysfunction causes a reduction β-cell mass and induces onset of diabetes [1]. Gene expression regulation among diabetic subjects with *HNF1*A variation can be explained by environmental factors together with epigenetic factors [22, 31].

This study had a case-control design and small sample size. *p.I27L* and *p.A98V* were consistent with the HWE whereas *p.S487* was not consistent with HWE. *HNF1A* gene *p.I27L* and *p.A98V* haplotypes were within LD. These are the limitations of this study.

## Conclusions

We report a genetic modifier of the *HNF1A* gene age at diagnosis that shows an effect of genetic variation on diabetes phenotype. The *HNF1A* variant *p.I27L* was associated with having early-onset, MODY-like diabetes in the Turkish population. Enlightening the role of *HNF1A* in β-cells would be helpful in understanding the molecular mechanism of both T2DM and MODY and would guide new therapeutic approaches.

## Additional file

Additional file 1: Genotyping and Statistical analysis. (DOCX 14 kb)

## Abbreviations

BMI: Body mass index
HbA_1c_: Hemoglobin A_1c_
HNF1A: Hepatocyte nuclear factor 1α
SNPs: Single nucleotide polymorphisms
T2DM: Type 2 diabetes mellitus

## Authors’ information

Selvihan Beysel MD, Nilnur Eyerci PhD, Ferda Alparslan Pinarli MD, Muhammed Kizilgul MD, Ozgur Ozcelik MD, Mustafa Caliskan MD, Erman Cakal MD.

## References

[1] Balamurugan K, Bjørkhaug L, Mahajan S, Kanthimathi S, Njølstad PR, Srinivasan N, Mohan V, Radha V. Structure-function studies of HNF1A (MODY3) gene mutations in South Indian patients with monogenic diabetes. Clin Genet. 2016;90:486–95. 10.1111/cge.12757 26853433

[2] Holmkvist J, Cervin C, Lyssenko V, Winckler W, Anevski D, Cilio C, Almgren P, Berglund G, Nilsson P, Tuomi T (2006). Common variants in HNF-1 alpha and risk of type 2 diabetes. Diabetologia.

[3] Weedon MN, Owen KR, Shields B, Hitman G, Walker M, McCarthy MI, Hattersley AT, Frayling TM (2005). A large-scale association analysis of common variation of the HNF1alpha gene with type 2 diabetes in the U.K. Caucasian population. Diabetes.

[4] Winckler W, Burtt NP, Holmkvist J, Cervin C, de Bakker PIW, Sun M, Almgren P, Tuomi T, Gaudet D, Hudson TJ (2005). Association of common variation in the HNF1alpha gene region with risk of type 2 diabetes. Diabetes.

[5] Scott RA, Lagou V, Welch RP, Wheeler E, Montasser ME, Luan J, Mägi R, Strawbridge RJ, Rehnberg E, Gustafsson S (2012). Large-scale association analyses identify new loci influencing glycemic traits and provide insight into the underlying biological pathways. Nat Genet.

[6] Cho YS, Chen C-H, Hu C, Long J, Ong RTH, Sim X, Takeuchi F, Wu Y, Go MJ, Yamauchi T (2012). Meta-analysis of genome-wide association studies identifies eight new loci for type 2 diabetes in east Asians. Nat Genet.

[7] Mahajan A, Go MJ, Zhang W, Below JE, Gaulton KJ, DIAbetes Genetics Replication And Meta-analysis (DIAGRAM) Consortium, Asian Genetic Epidemiology Network Type 2 Diabetes (AGEN-T2D) Consortium, South Asian Type 2 Diabetes (SAT2D) Consortium, Mexican American Type 2 Diabetes (MAT2D) Consortium, Type 2 Diabetes Genetic Exploration by Nex-generation sequencing in muylti-Ethnic Samples (T2D-GENES) Consortium (2014). Genome-wide trans-ancestry meta-analysis provides insight into the genetic architecture of type 2 diabetes susceptibility. Nat. Genet.

[8] Chiu KC, Chuang L-M, Chu A, Yoon C, Wang M (2003). Comparison of the impact of the I27L polymorphism of the hepatocyte nuclear factor-1alpha on estimated and measured beta cell indices. Eur. J. Endocrinol. Eur. Fed. Endocr. Soc..

[9] Urhammer SA, Fridberg M, Hansen T, Rasmussen SK, Møller AM, Clausen JO, Pedersen O (1997). A prevalent amino acid polymorphism at codon 98 in the hepatocyte nuclear factor-1alpha gene is associated with reduced serum C-peptide and insulin responses to an oral glucose challenge. Diabetes.

[10] Chiu KC, Chuang L-M, Chu A, Wang M (2003). Transcription factor 1 and beta-cell function in glucose-tolerant subjects. Diabet Med J Br Diabet Assoc.

[11] Morita K, Saruwatari J, Tanaka T, Oniki K, Kajiwara A, Otake K, Ogata Y, Nakagawa K (2015). Associations between the common HNF1A gene variant p.I27L (rs1169288) and risk of type 2 diabetes mellitus are influenced by weight. Diabetes Metab.

[12] Gaulton KJ, Ferreira T, Lee Y, Raimondo A, Mägi R, Reschen ME, Mahajan A, Locke A, Rayner NW, Robertson N (2015). Genetic fine mapping and genomic annotation defines causal mechanisms at type 2 diabetes susceptibility loci. Nat Genet.

[13] Bonnycastle LL, Willer CJ, Conneely KN, Jackson AU, Burrill CP, Watanabe RM, Chines PS, Narisu N, Scott LJ, Enloe ST (2006). Common variants in maturity-onset diabetes of the young genes contribute to risk of type 2 diabetes in Finns. Diabetes.

[14] Rissanen J, Wang H, Miettinen R, Kärkkäinen P, Kekäläinen P, Mykkänen L, Kuusisto J, Karhapää P, Niskanen L, Uusitupa M (2000). Variants in the hepatocyte nuclear factor-1alpha and -4alpha genes in Finnish and Chinese subjects with late-onset type 2 diabetes. Diabetes Care.

[15] Steenkamp DW, Alexanian SM, Sternthal E (2014). Approach to the patient with atypical diabetes. CMAJ Can Med Assoc J J Assoc Medicale Can.

[16] Ağladıoğlu SY, Aycan Z, Çetinkaya S, Baş VN, Önder A, Peltek Kendirci HN, Doğan H, Ceylaner S (2016). Maturity onset diabetes of youth (MODY) in Turkish children: sequence analysis of 11 causative genes by next generation sequencing. J Pediatr Endocrinol Metab JPEM.

[17] Gamboa-Meléndez MA, Huerta-Chagoya A, Moreno-Macías H, Vázquez-Cárdenas P, Ordóñez-Sánchez ML, Rodríguez-Guillén R, Riba L, Rodríguez-Torres M, Guerra-García MT, Guillén-Pineda LE (2012). Contribution of common genetic variation to the risk of type 2 diabetes in the Mexican mestizo population. Diabetes.

[18] Chen T, Cao X, Long Y, Zhang X, Yu H, Xu J, Yu T, Tian H (2010). I27L polymorphism in hepatocyte nuclear factor-1A gene and type 2 diabetes mellitus: a meta-analysis of studies about orient population (Chinese and Japanese). Int J Diabetes Mellit.

[19] Winckler W, Weedon MN, Graham RR, McCarroll SA, Purcell S, Almgren P, Tuomi T, Gaudet D, Boström KB, Walker M (2007). Evaluation of common variants in the six known maturity-onset diabetes of the young (MODY) genes for association with type 2 diabetes. Diabetes.

[20] Saxena R, Elbers CC, Guo Y, Peter I, Gaunt TR, Mega JL, Lanktree MB, Tare A, Castillo BA, Li YR (2012). Large-scale gene-centric meta-analysis across 39 studies identifies type 2 diabetes loci. Am J Hum Genet.

[21] Cauchi S, Nead KT, Choquet H, Horber F, Potoczna N, Balkau B, Marre M, Charpentier G, Froguel P, Meyre D (2008). The genetic susceptibility to type 2 diabetes may be modulated by obesity status: implications for association studies. BMC Med Genet.

[22] Bonatto N, Nogaroto V, Svidnicki PV, Milléo FQ, Grassiolli S, Almeida MC, Vicari MR, Artoni RF (2012). Variants of the HNF1A gene: a molecular approach concerning diabetic patients from southern Brazil. Genet Mol Biol.

[23] Deobagkar D, Ranade S, Deobagkar D (2010). Identification of I27L polymorphism in the HNF-1Agene in Western Indian population with late-onset of diabetes. Int J Diabetes Dev Ctries.

[24] Chiu KC, Chuang LM, Ryu JM, Tsai GP, Saad MF (2000). The I27L amino acid polymorphism of hepatic nuclear factor-1alpha is associated with insulin resistance. J Clin Endocrinol Metab.

[25] Bergman BC, Howard D, Schauer IE, Maahs DM, Snell-Bergeon JK, Eckel RH, Perreault L, Rewers M (2012). Features of hepatic and skeletal muscle insulin resistance unique to type 1 diabetes. J Clin Endocrinol Metab.

[26] Lehto M, Wipemo C, Ivarsson SA, Lindgren C, Lipsanen-Nyman M, Weng J, Wibell L, Widén E, Tuomi T, Groop L (1999). High frequency of mutations in MODY and mitochondrial genes in Scandinavian patients with familial early-onset diabetes. Diabetologia.

[27] Anuradha S, Radha V, Deepa R, Hansen T, Carstensen B, Pedersen O, Mohan V (2005). A prevalent amino acid polymorphism at codon 98 (Ala98Val) of the hepatocyte nuclear factor-1alpha is associated with maturity-onset diabetes of the young and younger age at onset of type 2 diabetes in Asian Indians. Diabetes Care.

[28] Yang Y, Zhou T-C, Liu Y-Y, Li X, Wang W-X, Irwin DM, Zhang Y-P (2016). Identification of HNF4A mutation p.T130I and HNF1A mutations p.I27L and p.S487N in a Han Chinese family with early-onset maternally inherited type 2 diabetes. J Diabetes Res.

[29] Yorifuji T, Higuchi S, Kawakita R, Hosokawa Y, Aoyama T, Murakami A, Kawae Y, Hatake K, Nagasaka H, Tamagawa N (2018). Genetic basis of early-onset, maturity-onset diabetes of the young-like diabetes in Japan and features of patients without mutations in the major MODY genes: dominance of maternal inheritance. Pediatr Diabetes.

[30] Locke JM, Saint-Martin C, Laver TW, Patel KA, Wood AR, Sharp SA, Ellard S, Bellanné-Chantelot C, Hattersley AT, Harries LW (2018). The common HNF1A variant I27L is a modifier of age at diabetes diagnosis in individuals with HNF1A-MODY. Diabetes.

[31] Awa WL, Thon A, Raile K, Grulich-Henn J, Meissner T, Schober E, Holl RW, DPV-Wiss. Study Group (2011). Genetic and clinical characteristics of patients with HNF1A gene variations from the German-Austrian DPV database. Eur J Endocrinol Eur Fed Endocr Soc.

