# Supplementary material for: HNF1A gene p.I27L is associated with early-onset, maturity-onset diabetes of the young-like diabetes in Turkey
Source: BMC Endocr Disord. 2019 May 20;19:51. doi: 10.1186/s12902-019-0375-2 (PMC6528345; doi:10.1186/s12902-019-0375-2)
Supplement: Supplementary file 1 — Genotyping and Statistical analysis. (DOCX 14 kb) [file 12902_2019_375_MOESM1_ESM.docx]

*Genotyping*

Genomic DNA was isolated from collected whole blood samples using the High Pure PCR Template Preparation Kit (Roche Life Science, Indianapolis, USA). All exons, the proximal promoter regions, and exon-intron boundaries of four genes, *HNF1A*, *GCK*, *HNF4A* and *HNF1B*, were amplified by polymerase chain reaction (PCR), with the use of specific primer sets. Polymerase chain reaction (PCR) was performed in 20 μL final volume containing 20 ng of genomic DNA, 1 μL of each primer, 12,5 μL PCR Master Mix (GML AG, Altendorf, Switzerland), 0.2 μL Taq DNA polymerase (GML AG, Altendorf, Switzerland). The PCR reaction was initiated by denaturing the sample at 96°C for 5 min followed by 40 cycles of denaturation at 94 °C for 30 s, annealing at 60°C for 45 s and extension at 72 °C for 45 s. Final extension was done at 72°C for 10min. All the reactions were performed in ABI Veriti Thermal Cycler (Applied Biosystems, Foster City, CA, USA). PCR products were cleaned up using ExoSAP-IT reagent (GML AG, Altendorf, Switzerland). Then, sequencing reaction was performed, on both strands, by BigDye Terminator v3.1 Cycle Sequencing Kit (Applied Biosystems, Foster City, CA, USA) in ABI Veriti thermocycler. Sequencing products were purified by Applied Biosystems BigDye XTerminator Purification Kit (Applied Biosystems, Foster City, CA, USA), then, capillary electrophoresis performed on 3130xl Genetic Analyzer (Applied Biosystems, Foster City, CA, USA). Sequences were analysed using softwares Seqscape v3.1 and variant reporter v.1 (Applied Biosystems, Foster City, CA, USA).

Genetic analyses for *HNF1A* gene *p.S487N* (rs2464196), *p.A98V* (rs1800574) and *p.I27L* (rs1169288) SNPs were performed by Sanger-based DNA sequencing among control group. Genotyping of each *HNF1A* gene polymorphisms were independently carried out using prevalidated a fluorescence-based allele-specific PCR assay, KASPar (KBiosciences, Hoddesdon, UK), performed on a Rotor-Gene Q real-time cycler (Qiagen, Hilden, Germany) according to the manufacturer’s instructions. Allele discrimination was made using Rotor-Gene Q software v.2.3.1 (Qiagen, Hilden, Germany). The genotype calling was performed blind without information on clinical phenotypes.

*Statistical analysis*

Statistical analysis was performed using SPSS 18.0 (SPSS, Inc) soft-ware. Variables are presented as mean±standard deviation (SD) or median (min-max), percentages (%), odds ratios (*OR*), 95 % confidence intervals (*CI*). Normality was tested using the Kolmogorov-Smirnov and Shapiro-Wilk *W* test. Density of SNPs was expressed as allelic frequency (q) or prevalence of genotypes. Categorical variables were analyzed with the Chi-square test or Fisher’s exact test, where appropriate. Mann-Whitney U test was used for continuous variables without normally distributed. Student’s *t* test was used for normally distributed continuous variables or log-transformed variables between two groups. The Hardy-Weinberg equilibrium (HWE) at individual loci was assessed by Chi-Square test. Correlation analysis between dependent (diabetes) and independent variants (SNPs) were tested by Spearman’s correlation. Multivariate analyses were performed for adjusting BMI. Pair-wise linkage disequilibrium (LD) and correlation coefficients (r^2^) were analyzed using the HAPLOVİEW program. We made a variable reflecting all possible combinations of *p.I27L, p.A98V* and *p.S487N* genotypes for each SNP. This study was recruited 749 subjects to have 68 % power with % 5 type 1 error level to detect a minimum clinically significant difference. The power analysis was performed according to <http://osse.bii.a-star.edu.sg/calculation2.php>. Statistical significance was defined as a *p* < 0.05.
